# Ulipristal acetate vs gonadotropin‐releasing hormone agonists prior to laparoscopic myomectomy (MYOMEX trial): Short‐term results of a double‐blind randomized controlled trial

**DOI:** 10.1111/aogs.13713

**Published:** 2019-09-27

**Authors:** Inge de Milliano, Judith A. F. Huirne, Andreas L. Thurkow, Celine Radder, Marlies Y. Bongers, Huib van Vliet, Jonas van de Lande, Peter M. van de Ven, Wouter J. K. Hehenkamp

**Affiliations:** ^1^ Department of Obstetrics and Gynecology Amsterdam Reproduction and Development University Medical Center Amterdam, location VU Medical Center Amsterdam The Netherlands; ^2^ Department of Obstetrics and Gynecology University Medical Center Amterdam location Academic Medical Center Amsterdam The Netherlands; ^3^ Department of Obstetrics and Gynecology OLVG Amsterdam The Netherlands; ^4^ Department of Obstetrics and Gynecology Maxima Medical Center Veldhoven The Netherlands; ^5^ Department of Obstetrics and Gynecology GROW School for Oncology and Developmental Biology Maastricht University Medical Center Maastricht The Netherlands; ^6^ Department of Obstetrics and Gynecology Catharina Hospital Eindhoven The Netherlands; ^7^ Department of Obstetrics and Gynecology Spaarne Gasthuis Haarlem The Netherlands; ^8^ Department of Biostatistics VU University Amsterdam The Netherlands

**Keywords:** gonadotropin‐releasing hormone agonist, intraoperative blood loss, laparoscopic myomectomy, pretreatment, surgical ease, ulipristal acetate

## Abstract

**Introduction:**

Laparoscopic myomectomy can be difficult when fibroids are large and numerous. This may result in extensive intraoperative bleeding and the need for a conversion to a laparotomy. Medical pretreatment prior to surgery might reduce these risks by decreasing fibroid size and vascularization of the fibroid. We compared pretreatment with ulipristal acetate (UPA) vs gonadotropin‐releasing hormone agonists (GnRHa) prior to laparoscopic myomectomy on several intra‐ and postoperative outcomes.

**Material and methods:**

We performed a non‐inferiority double‐blind randomized controlled trial in nine hospitals in the Netherlands. Women were randomized between daily oral UPA for 12 weeks and single placebo injection or single intramuscular injection with leuprolide acetate and daily placebo tablets for 12 weeks. The primary outcome was intraoperative blood loss. Secondary outcomes were reduction of fibroid volume, suturing time, total surgery time and surgical ease.

**Results:**

Thirty women received UPA and 25 women leuprolide acetate. Non‐inferiority of UPA regarding intraoperative blood loss was not demonstrated. When pretreated with UPA, median intraoperative blood loss was statistically significantly higher (525 mL [348‐1025] vs 280 mL[100‐500]; *P *=* *0.011) and suturing time of the first fibroid was statistically significantly longer (40 minutes [28‐48] vs 22 minutes [14‐33]; *P *=* *0.003) compared with GnRHa. Pretreatment with UPA showed smaller reduction in fibroid volume preoperatively compared with GnRHa (−7.2% [−35.5 to 54.1] vs −38.4% [−71.5 to −19.3]; *P *=* *0.001). Laparoscopic myomectomies in women pretreated with UPA were subjectively judged more difficult than in women pretreated with GnRHa.

**Conclusions:**

Non‐inferiority of UPA in terms of intraoperative blood loss could not be established, possibly due to the preliminary termination of the study. Pretreatment with GnRHa was more favorable than UPA in terms of fibroid volume reduction, intraoperative blood loss, hemoglobin drop directly postoperatively, suturing time of the first fibroid and several subjective surgical ease parameters.

**Abbreviations**
GnRHa: gonadotropin‐releasing hormone agonist
UPA: ulipristal acetate

**Key message**

Non‐inferiority of ulipristal acetate in terms of intraoperative blood loss could not be established. Pretreatment with gonadotropin‐releasing hormone agonist seems to be more favorable than ulipristal acetate for several operative outcomes and subjective surgical parameters.

## 1 INTRODUCTION

Laparoscopic myomectomy seems to have several advantages over the laparotomic approach. Smaller incisions result in less postoperative pain, shorter hospital stay and faster recovery.^1^, ^2^, ^3^ However, laparoscopic myomectomy can be difficult when fibroids are large and numerous. This may result in extensive intraoperative bleeding and the need for a conversion to a laparotomy. Medical pretreatment prior to surgery might reduce these risks by decreasing fibroid size and vascularization of the fibroid.

Only two medications are registered for the pretreatment of fibroids. Gonadotropin‐releasing hormone agonists (GnRHa) are considered the gold standard. Pretreatment with GnRHa improves pre‐ and postoperative hemoglobin level and reduces uterine and fibroid volume.^4^

Ulipristal acetate (UPA), a selective progesterone receptor modulator, has recently been approved for preoperative treatment of uterine fibroids. UPA has pro‐apoptotic and anti‐proliferative effects on the fibroid and normal myometrial tissue remains unaffected.

No randomized trials are available reporting on surgical outcomes comparing pretreatment with GnRHa or UPA. In this double‐blind randomized controlled trial, we evaluate whether pretreatment with UPA was non‐inferior to pretreatment with GnRHa (11.25 mg) on intraoperative and postoperative outcomes of laparoscopic myomectomy.

## 2 MATERIAL AND METHODS

### 2.1 Study design

We performed a double‐blind randomized controlled trial in nine hospitals in the Netherlands comparing UPA and GnRHa prior to laparoscopic myomectomy. Participating hospitals were selected on extensive experience (>150 per year) with level 3 and 4 gynecological laparoscopic surgery as defined by Royal College of Obstetricians and Gynaecologists.

### 2.2 Study population

Premenopausal women for a laparoscopic resection of a maximum of two FIGO (PALM‐COEIN classification) type 3, 4, 5, 6, or two to five uterine fibroids with a diameter of 5‐12 cm were eligible for participation in this study. Exclusion criteria were age below 18 years, pregnancy, suspicion of malignancy, use of hormonal agents, chronic use of anticoagulants, coagulopathy, contraindication to laparoscopic procedures or allergy to leuprolide acetate or UPA.

### 2.3 Randomization and treatment

Eligible women visiting the outpatient clinic of one of the participating centers were informed about the study by their gynecologist. After written informed consent was given, participating women were randomly allocated in a one‐to‐one ratio to receive either GnRHa or UPA. Randomization was performed using a computer‐generated randomization system and stratified by center. Women in the GnRHa group received a single intramuscular injection of leuprolide acetate (11.25 mg in 1 mL) and daily placebo tablets for 12 consecutive weeks. Participants in the UPA group received daily oral UPA 5 mg for 12 consecutive weeks and a one‐time placebo injection containing 1 mL saline. Study materials and medication packaging were identical for both groups. Treatment was preferably started in the first week of the menstruation period. Surgery was performed within a month after the last tablet. Participants and gynecologists were blinded to treatment allocation during the entire study period. Statistics were performed by an independent statistician blinded to the allocated study groups.

### 2.4 Outcome measures

The primary outcome was intraoperative blood loss. Secondary outcomes were time of surgery, time of enucleation, time of suturing, surgical ease and reduction in fibroid volume. For a careful explanation of all outcome measures, see Appendix S1.

### 2.5 Laparoscopic myomectomy

Surgery was performed by experienced surgeons. The procedure was performed under general anesthesia after administration of prophylactic broad‐spectrum antibiotics. An expert meeting of participants was held on 2 June 2014 to reach consensus on the surgical technique. Relevant surgical characteristics were divided in: “standard use”, “never use” or “optional use”, defined as: Standard use—use of barbed sutures, use of (any) uterine manipulator, use of blue dye in uterine cavity in order to diagnose whether the cavity was opened or not. For fibroids >8 cm it was allowed to apply bulldogs on the uterine artery and infundibulopelvic ligament. Never use—vasoconstrictive medication such as glypressin or use of bulldogs for fibroids <8 cm. Optional use—single administration of 1000 mg of tranexamic acid or use of hemostatic or anti‐adhesive products on uterine incision were allowed only after operative blood loss has been calculated.

### 2.6 Statistical analyses

The trial was a non‐inferiority trial with the following null hypothesis: UPA is non‐inferior to GnRHa in terms of blood loss during surgery with a maximum difference of 150 mL considered acceptable based on previous studies on this subject.^5^ The assumed standard deviation was 250 mL for intraoperative blood loss based on a survey in three hospitals in the Netherlands. Based on a two‐group *t* test of equivalence in means, using an one‐sided significance level of 2.5% (one‐sided), and a Type II error of 20% (80% power) this yields a sample size of 90 women (45 in each study arm).

The analyses of the primary outcome intraoperative blood loss were performed both according to the per protocol principle and intention‐to‐treat principle. All other analyses were performed according to the intention‐to‐treat principle. Normality of the data was assessed visually by means of QQ plots. Because the primary outcome itself did not appear to be normally distributed, but became normally distributed after a log‐transformation, we used the following procedure for testing non‐inferiority. To take into account the non‐inferiority margin of 150 mL defined on the original scale, we added 150 mL to the observed blood losses for GnRHa but left observed blood losses for UPA unchanged. This was done to create a setting in which the difference of the log‐transformation of the adapted blood loss was 0 exactly if the blood loss in UPA pretreated women is 150 mL higher than pretreatment with GnRHa (the null hypothesis of the non‐inferiority test). Non‐inferiority was concluded if the confidence interval for the differences in means for the log‐transformation of these adapted outcomes lay entirely below 0 mL.

Normally distributed data were summarized as mean ± standard deviation and were compared with the independent *t* test. For continuous outcomes that were not normally distributed we present median and interquartile range. Depending on the exact distribution, we used an independent sample *t* test on the log‐transformed outcome or the Mann‐Whitney *U* test when comparing the outcomes between the groups. The Mann‐Whitney test was also used for comparison of ordinal variables between the groups. Dichotomous and categorical outcomes were summarized by frequencies and percentage. Chi‐square test and Fisher's exact test were used to compare the distribution of these outcomes between groups.

To assess differences between baseline and after 3 months within a group, we used the paired *t* test for normally distributed variables, the Wilcoxon signed rank test for non‐normally distributed data and the McNemar test for dichotomous variables.

Linear regression analyses were performed to correct the analyses for comparison of mean blood loss and total surgery time between pretreatment groups for potential confounders. A variable was considered a confounder when the regression coefficient for groups changed by >10% when the confounder was added to the model. Potential confounders with a skewed distribution were log‐transformed to decrease of the impact of outliers.

All analyses were performed using SPSS version 22.0 (IBM Corp., Armonk, NY, USA). Non‐inferiority of blood loss was tested at a one‐sided significance level of 2.5%. A two‐sided significance level of 5% was used for all other analyses.

### 2.7 Ethical approval

This study was approved by the National Central Committee on Research Involving Human Subjects (CCMO ‐ NL49916.029.14), by the ethics committee of VU Medical Center Amsterdam (Ref. No. 2014/421, date 17‐12‐2014) and by the boards of all participating hospitals. The trial protocol has been registered on ClinicalTrials.gov (NCT02288130).

## 3 RESULTS

### 3.1 Women

**Figure 1:**
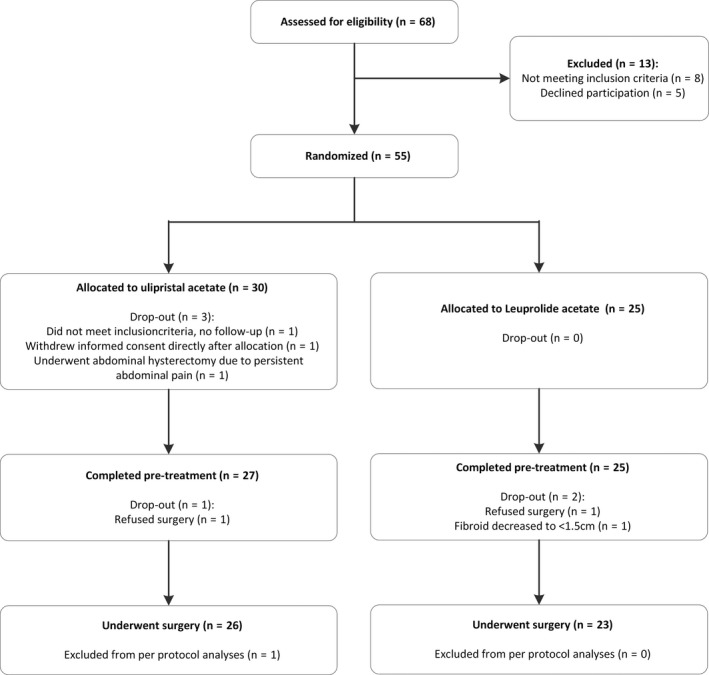
CONSORT flow diagram

Women were enrolled between May 2015 and July 2017. Due to disappointing inclusion rates in most participating centers and expiration of study medication, the intended number of inclusions was not met. Six of nine participating hospitals included women (Table S1). Of 68 eligible women, 55 were randomized: 30 allocated to UPA and 25 to leuprolide acetate (Figure 1). One woman randomized to UPA in retrospect did not meet the inclusion criteria (fibroid >12 cm) and was excluded from analysis. In the UPA group, two women dropped out before the end of pretreatment; one woman withdrew informed consent directly after allocation, so no follow up occurred and one woman underwent abdominal hysterectomy 6 weeks after start of medication due to persistent severe abdominal pains and fibroid growth. In the GnRHa group, all women completed pretreatment. A total of three women did not undergo a laparoscopic myomectomy. Two women (one in each group) refused surgery because they preferred homeopathic therapy. For one woman allocated GnRHa, surgery was cancelled due to major decrease in fibroid volume from 80 cm^3^ to 1 cm^3^.

The two treatment groups were similar in demographic characteristics and hemoglobin level before pretreatment (Table 1). However, treatment groups differed in fibroid characteristics, due to lack of stratification for fibroid size at baseline. The mean diameter of the largest fibroid was significantly higher in women allocated UPA than women allocated GnRHa (8.5 ± 1.9 vs 7.4 ± 1.6 cm; *P *=* *0.035; 95% CI .1‐2.0). Other fibroid characteristics at baseline (ie, type, uterine volume and total fibroid volume planned for resection) did not show any significant difference.

**Table 1:** Baseline characteristics of the study population

	Ulipristal acetate (n = 29)	leuprolide acetate (n = 25)	*P* value
Age (years; mean ± SD)	38.4 ± 5.8	41.4 ± 5.6	0.058
Body mass index (kg/m^2^; median, range)	24.5 (18.8‐38.2)	25.9 (18.0‐42.5)	0.466
Parity (median, range)	0 (0‐2)	1 (0‐4)	0.112
Race (n; %)			
Caucasian	17 (59)	11 (44)	0.357
Black	3 (10)	6 (24)	
Other	9 (31)	8 (32)	
Medical history of abdominal surgery (n, %)	14 (48)	8 (32)	0.225
Hemoglobin (mmol/L; mean ± SD)	7.8 ± 1.0	7.7 ± 1.1	0.742
Indication for surgery (n)^a^			
Heavy menstrual bleeding	11	13	NA
Abdominal pain	9	8	
Subfertility	8	2	
Mechanical complaints^b^	15	7	
Other	2	1	
Type of largest fibroid^c^ (n, %)			
3	—	3 (12)	0.093
4	1 (3)	2 (8)	
5	4 (14)	4 (16)	
6	9 (31)	3 (12)	
2‐5	15 (52)	13 (52)	
Mean diameter of largest fibroid (cm; mean ± SD)	8.5 ± 1.9	7.4 ± 1.6	**0.035**
Total fibroid volume planned for resection (cm^3^; median, IQR)	316.3 (184.7‐462.6)	246.0 (130.2‐344.3)	0.094
Uterine volume (cm^3^; median, IQR)	530.5 (392.8‐774.5)	421.2 (327.5‐819.8)	0.450

Abbreviations: IQR, interquartile range; SD, standard deviation.

Bold indicates statistically significant values.

a Patients could have more than one indication for surgery.

b Eg, constipation, urinary retention.

c Type of fibroid following the FIGO (PALM‐COEIN) classification.

Compliance to study medication was high in both groups. Only two women (one in each group) reported that they forgot to take their oral study medication daily (UPA group 50 tablets remaining; GnRHa group 8 tablets remaining). For the per protocol analyses of the primary outcome, the woman who did not comply with UPA was excluded from analyses.

### 3.2 Intraoperative blood loss

As intraoperative blood loss was not normally distributed, non‐inferiority could not be assessed in the standard manner using the 95% CI for the differences in mean blood loss. The alternative approach using a log‐transformation of the adapted outcomes as described in the statistical analysis yielded a 95% CI for which the upper bound exceeded 0 (95% CI for difference −0.06 to 0.30 for both per protocol and intention to treat analyses) and hence this trial does not support the alternative hypothesis that UPA is non‐inferior to GnRHa. After correction for confounder mean diameter of the largest fibroid, the 95% confidence interval for the mean difference of the logs still contained 0 (per protocol analyses: 95% CI for difference −0.20 to 0.13; intention‐to‐treat analyses: 95% CI for difference −0.20 to 0.14), which did not alter the conclusion.

Pretreatment with UPA results in significant higher median intraoperative blood loss compared with pretreatment with GnRHa (525 mL [interquartile range 348‐1025; range 100‐2275] vs 280 mL] 100‐500; range 40‐2200], *P *=* *0.002) (Table 2). Number of fibroids removed, fibroid type and mean diameter of largest fibroid at baseline were tested for potential confounding using linear regression analysis. Only mean diameter of the largest fibroid at baseline appeared to be a confounder. Correction for this confounder resulted in a *P* value of 0.011.

**Table 2:** Results on intraoperative outcomes

	Ulipristal acetate (n = 26)	leuprolide acetate (n = 23)	*P* value
Intraoperative blood loss (mL; median, IQR)	525 (348‐1025)	280 (100‐500)	**0.002** a ^**,**^ b
Total surgery time (min; median, IQR)	188 (132‐231)	125 (100‐175)	**0.023** a ^**,**^ c
Time of enucleation (min; median, IQR)	51 (31‐86)	35 (26‐66)	0.203
Fibroid 1	49 (31‐86)	33 (25‐60)	0.191
Fibroid 2	10 (5‐15)	8 (5‐25)	0.916
Suturing time (min; median, IQR)	42 (29‐51)	25 (14‐40)	**0.009**
Fibroid 1	40 (28‐48)	22 (14‐33)	**0.003**
Fibroid 2	5 (3‐40)	18 (7‐30)	0.325
Time of morcellation (min; median, IQR)	13 (5‐25)	7 (4‐14)	0.085
Number of fibroids removed (n; %)			
1	21 (81)	19 (83)	0.868
≥2	5 (19)	4 (17)	
Weight of fibroids removed (g; median, IQR)	349 (185‐561)	140 (61‐272)	**0.001**
Hemoglobin drop (mmol/L; mean ± SD)	1.8 ± 1.3	1.0 ± 0.8	**0.012**
Bulldogs on uterine artery (n, %)	4 (15)	2 (9)	0.671
Bulldogs on ovarian vessels (n, %)	10 (39)	3 (13)	**0.044**
Opening of cavum (n, %)	4 (15)	5 (22)	0.716
Conversion rate (n, %)	3 (12)	0 (0)	0.237
Complication rate (n, %)^d^	4 (15)	2 (9)	0.671

Abbreviations: IQR, interquartile range; SD, standard deviation.

Bold indicates statistically significant values.

a Unpaired *t* test performed on log‐transformed variable intraoperative blood loss and total surgery time.

b After correction for confounder ‘mean diameter of largest fibroid’: *P *=* *0.011.

c After correction for confounder ‘mean diameter of largest fibroid’: *P *=* *0.053.

d All intraoperative complications in both groups were blood loss >1 L.

Hemoglobin level within 48 hours of surgery compared with hemoglobin level before surgery was statistically significantly lower in the UPA group compared with GnRHa (−1.8 ± 1.3 vs −1.0 ± 0.8 mmol/L; 95% CI for difference 0.2‐1.4; *P *=* *0.012).

### 3.3 Secondary outcomes

#### 3.3.1 Differences from baseline to 3 months

Table 3 shows changes in fibroid characteristics and hemoglobin levels between baseline and after 3 months of pretreatment. Change in mean diameter of the largest fibroid was found to differ between pretreatment with UPA and pretreatment with GnRHa (−3.6% [−15.5 to 10.4] vs −14.6% [−40.7 to −5.6]; *P *=* *0.003). Total reduction in volume of the fibroids that were planned for resection was statistically significantly less after UPA than after GnRHa pretreatment (−7.2% [−35.5 to 54.1] vs −38.4% [−71.5 to −19.3]; *P *=* *0.001). Reduction in uterine volume after 3 months was also statistically significantly smaller in the UPA group than with GnRHa (−6.4% [−24.3 to 51.0] vs −26.2% [−63.4 to 4.2]; *P *=* *0.020). Hemoglobin levels increased significantly in both pretreatment groups during pretreatment (Table 2).

**Table 3:** Changes in characteristics of fibroids and hemoglobin levels after 3 months’ pretreatment

	Ulipristal acetate (n = 29)	leuprolide acetate (n = 25)	*P* value
Mean diameter of largest fibroid (cm; mean ± SD)			
Baseline	8.5 ± 1.9	7.4 ± 1.6	
3 months	8.4 ± 2.3	5.8 ± 2.1^a^	
Change from baseline to 3 months in cm	−0.1 ± 1.7	−1.6 ± 1.5	**0.002**
Change from baseline to 3 months in % (median, IQR)	−3.6% (−15.5 to 10.4)	−14.6% (−40.7 to −5.6)	**0.003**
Total fibroid volume planned for resection (cm^3^; median, IQR)			
Baseline	316.3 (184.7‐462.6)	246.0 (130.2‐344.3)	
3 months	319.4 (163.0‐506.4)	105.9 (54.4‐195.9)^b^	
Change from baseline to 3 months in cm^3^	−33.0 (−109.5 to 95.8)	−80.2 (−183.6 to −33.2)	**0.012**
Change from baseline to 3 months in %	−7.2% (−35.5 to 54.1)	−38.4% (−71.5 to −19.3)	**0.001**
Uterine volume (cm^3^; median, IQR)			
Baseline	530.5 (392.8‐774.5)	421.2 (327.5‐819.8)	
3 months	598.2 (284.6‐830.6)	272.1 (180.4‐508.8)^c^	
Change from baseline to 3 months in cm^3^	−28.1 (−161.2 to 107.3)	−151.5 (−256.6 to 9.4)	0.081
Change from baseline to 3 months in %	−6.4% (−24.3 to 51)	−26.1% (−63.4 to 4.2)	**0.020**
Hemoglobin (mmol/L, mean ± SD)			
Baseline	7.8 ± 1.0	7.7 ± 1.1	
3 months	8.4 ± .8^d^	8.2 ± 0.9^d^	
Change from baseline to 3 months	0.5 ± 0.9	0.5 ± 0.9	0.859

Abbreviations: IQR, interquartile range; SD, standard deviation.

Bold indicates statistically significant values.

a Statistically significant compared with baseline (*P *<* *0.001).

b Statistically significant compared with baseline (*P *<* *0.001).

c Statistically significant compared with baseline (*P *=* *0.015).

d Statistically significant compared with baseline (ulipristal acetate *P *=* *0.012; GnRHa *P *=* *0.013).

#### 3.3.2 Other intraoperative outcomes

In 81% of women pretreated with UPA, only one fibroid was removed, compared with 83% of women pretreated with GnRHa (*P *=* *0.868). Unadjusted analysis of total surgery time showed a longer surgery time in the UPA group compared with GnRHa (188 minutes [132‐231] vs 125 minutes [100‐175]; *P *=* *0.023) (Table 2). A potential confounding effect on surgery time of number of fibroids removed and the mean diameter of the largest fibroid at baseline was tested. Only mean diameter of the largest fibroid at baseline appeared to be a confounder. After correction for this confounder, the difference in surgery time between both groups did not reach statistical significance (*P* = 0.053). No difference in time of enucleation and time of morcellation was found between both groups. Suturing time for the largest fibroid was longer in the UPA group (40 minutes [28‐48] vs 22 minutes [14‐33]; *P *=* *0.003). The weight of the fibroids removed was significantly higher in women pretreated with UPA (349 g [185‐561] vs 140 g [61‐272]; *P *=* *0.001).

In women pretreated with UPA, the ovarian vessels were clamped more frequently than in the GnRHa group (10 times vs 3 times; *P *=* *0.044) because in 52% of the women in the UPA group, the mean diameter of the largest fibroid remained >8 cm despite pretreatment. In the GnRHa group, this was 16% (*P *=* *0.004). No difference was found in frequency of clamping of the uterine artery, opening of the uterine cavum or conversion rate. Six intraoperative complications were reported, four in the UPA group and two in the GnRHa group (all intraoperative blood loss of >1 L).

#### 3.3.3 Surgical ease

Most items of the surgical assessment tool show a significant difference between both treatment groups (Table 4). Procedures of women pretreated with UPA were found to be more difficult than procedures of women pretreated with GnRHa (4 [3.0‐5.0] vs 3 [2.0‐4.0]; *P *=* *0.011). Surgeons in the UPA group were less satisfied compared with those in the GnRHa group (2 [2.0‐3.3] vs 2 [1.0‐2.0] *P *=* *0.027). Surgeons found it more difficult to identify cleavage planes in women pretreated with UPA than with GnRHa (4 [2.7‐4.0] vs 3 [1.0‐4.0]; *P *=* *0.035). They also reported more difficulties with morcellation of fibroids pretreated with UPA compared with GnRHa (3 [2.0‐3.0] vs 2 [2.0‐3.0]; *P *=* *0.011). In women pretreated with UPA, surgeons reported softer fibroids than in women pretreated with GnRHa (2 [1.0‐3.0] vs 3 [2.0‐4.0]; *P *=* *0.017). This resulted in a poorer grip on the fibroid during surgery in the UPA group (2 [1.0‐3.0] vs 1 [1.0‐2.0]; *P *=* *0.001). Stitching the myometrium was assessed as more difficult in women who received UPA than GnRHa (3 [3.0‐3.0] vs 3 [2.0‐3.0]; *P *=* *0.011). The subjectively evaluated bleeding tendency of the tissue at surgery was reported to be higher in the UPA group than the GnRHa group (3 [3.0‐4.0] vs 3 [2.0‐3.0]; *P *=* *0.004). The grip of barbed sutures in the myometrium and the anatomical result did not differ between the treatment groups.

**Table 4:** Results on surgical ease

		1	2	3	4	5	*P* value
Difficulty of entire procedure (median, IQR) a *1 = very easy; 2 = easy; 3 = moderate; 4 = difficult; 5 = very difficult*	UPA^b^	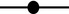					**0.011**
	GnRHa^c^	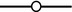					
Satisfaction with entire procedure (median, IQR) *1 = very satisfied; 2 = satisfied; 3 = moderate; 4 = unsatisfied; 5 = very unsatisfied*	UPA	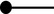					**0.027**
	GnRHa	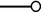					
Difficulty finding cleavage planes fibroid/capsula (median, IQR) *1 = very easy; 2 = easy; 3 = moderate; 4 = difficult; 5 = very difficult*	UPA	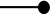					**0.035**
	GnRHa	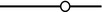					
Difficulty of morcellation (median, IQR) *1 = very easy; 2 = easy; 3 = moderate; 4 = difficult; 5 = very difficult*	UPA	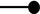					**0.011**
	GnRHa	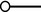					
Consistency of fibroid (median, IQR) *1 = very soft; 2 = soft; 3 = normal; 4 = firm; 5 = very firm*	UPA	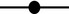					**0.017**
	GnRHa	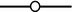					
Grip on fibroid (median, IQR) *1 = good; 2 = moderate; 3 = bad*	UPA	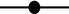					**0.001**
	GnRHa	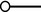					
Grip of barbed sutures in myometrium (median, IQR) *1 = good; 2 = moderate; 3 = bad*	UPA	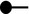					0.151
	GnRHa	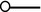					
Ease of stitching (median, IQR) *1 = very easy; 2 = easy; 3 = moderate; 4 = difficult; 5 = very difficult*	UPA	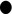					**0.011**
	GnRHa	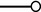					
Bleeding tendency of tissue at surgery (median, IQR) *1 = almost none; 2 = little; 3 = normal; 4 = more than average; 5 = very bloody*	UPA	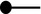					**0.004**
	GnRHa	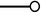					
Result anatomically (median, IQR) *1 = very satisfied; 2 = satisfied; 3 = moderate; 4 = unsatisfied; 5 = very unsatisfied*	UPA	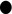					0.054
	GnRHa	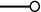					

a IQR = interquartile range.

b UPA = ulipristal acetate (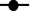).

c GnRHa = Gonadotropin Releasing‐hormone Agonist (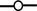).

#### 3.3.4 Side‐effects, postoperative complications and serious adverse events

To assess the prevalence of the most common side‐effects, headaches and hot flushes, the Menopause questionnaire by Oldenhave et al^6^ was used. The number of women reporting moderate to severe headaches after 3 months was not significantly higher compared with baseline in both groups (UPA: 36 vs 32%; GnRHa: 16 vs 35%). The number of women experiencing moderate to severe hot flushes was significantly higher after 3 months for both groups (UPA: 7 vs 43%, *P *=* *0.006; GnRHa 12 vs 65%, *P* = 0.004). The frequency of hot flushes after 3 months did not differ between groups (*P *=* *0.111).

A total of six postoperative complications were reported, four in the UPA group and two in the GnRHa group. For detailed information on these complications see Appendix S2.

## 4 DISCUSSION

In this double‐blinded randomized controlled trial, non‐inferiority of UPA to GnRHa in terms of intraoperative blood loss could not be established (using a predefined non‐inferiority margin of 150 mL). This can be explained by the limited sample size; however, it can not be excluded that UPA is inferior to GnRHa as a pretreatment for laparoscopic myomectomy. Median intraoperative blood loss was 245 mL higher in the group pretreated with UPA than GnRHa. This was in line with the higher hemoglobin drop postoperatively in women pretreated with UPA. Mediation analysis showed that the difference in intraoperative blood loss between both groups can partly be explained by less heavy fibroids after pretreatment with GnRHa and lower fibroid weight, in turn associated with lower intraoperative blood loss (Appendix S3). Suturing time of the first fibroid is significantly longer in women who received UPA as pretreatment compared with GnRHa. This could explain the higher intraoperative blood loss in the UPA group or the larger fibroid size at the time of surgical removal. Within the confines of this trial, pretreatment with UPA results in a significantly smaller decrease in fibroid volume and uterine volume compared with GnRHa. Also, laparoscopic myomectomies in women pretreated with UPA were subjectively evaluated as more difficult than in women pretreated with GnRHa.

No previous studies have been published comparing UPA with GnRHa prior to laparoscopic myomectomy. Previous randomized trials compared GnRHa with placebo or immediate surgery before laparoscopic myomectomy.^7^, ^8^, ^9^ No randomized trials have been performed comparing UPA with placebo before laparoscopic myomectomy. Assumptions on intraoperative blood loss made in designing this study are based on a systematic review and meta‐analysis by Chen et al^5^ comparing pretreatment with GnRHa with no pretreatment. Mean intraoperative blood loss in the included studies (n = 3) for women pretreated with GnRHa varied from 172 to 199 mL. This is lower than the median intraoperative blood loss found in our study of 280 mL in women pretreated with GnRHa. This can be explained by smaller fibroid diameter or fibroid volume of the previous studies (ie, volume of largest fibroid is ±65 cm^3^ in these studies, whereas median volume of fibroid planned for resection in our study is 246 cm^3^). The predefined non‐inferiority margin of 150 mL may have been too small, considering the larger fibroids included in our study. This could be why non‐inferiority was not established.

A randomized trial by Donnez et al^10^ comparing UPA with GnRHa, did not show significant differences in reduction of fibroid volume after 3 months of pretreatment between the two groups. The difference with our study may be explained by the size of the fibroids included in these studies. In the study by Donnez et al, the median cumulative volume of the three largest fibroids at baseline is 79.6 cm^3^ in the UPA group and 59.2 cm^3^ in the leuprolide acetate group. These fibroids are much smaller than the total fibroid volume planned for resection in our study (ie, 316.3 cm^3^ and 246.0 cm^3^, respectively, at baseline).

Well conducted studies on surgical ease in pretreated women undergoing laparoscopic myomectomy are very limited. Only two retrospective studies have been published on this subject comparing surgical experience in women pretreated with UPA, with no hormonal pretreatment prior to myomectomy^11^, ^12^ showing overall no difference in surgical experience.

This is the first randomized controlled trial performed on intraoperative outcomes comparing UPA with GnRHa prior to laparoscopic myomectomy. This trial was performed double‐blind, resulting in women and surgeons who were unaware of the pretreatment received. This is particularly important for subjective outcomes such as surgical ease. An important limitation of this study is that the anticipated total of 90 women could not be recruited due to expiration of study medication and disappointing inclusion rates. These can be explained by physician preferences for one of the treatments, an overestimation of most participating centers of the number of laparoscopic myomectomies they perform on a yearly basis, and the fact that many eligible women were already (pre)treated with UPA or GnRHa in another hospital before they were referred to one of the participating centers. Despite this, several outcomes reached statistical significance. It is not possible to conclude whether some of these significant differences were caused by chance (type I error). Another limitation is the fact that we did not stratify for fibroid size at baseline, resulting in an unbalanced distribution of the fibroid size in both groups. A regression analysis was performed to correct for this confounder; however, it cannot be excluded that this difference at baseline had a subsequent effect on many of the other endpoints such as blood loss and weight of fibroids. Additionally, we present the stratified results of fibroids ≤8 cm or >8 cm (Table S2). The direction and trend of the differences between UPA and GnRHa remain the same.

The majority of women were included in one center. Sensitivity analyses did not show differences in intraoperative blood loss for this center compared with other centers (Table S1). An additional limitation of the study may be that the questionnaire to assess surgical ease is non‐validated due to very limited studies on this subject, so a total score could not be given. Surgeons were blinded to the pretreatment received in both treatment arms and each question should be interpreted as an individual item without calculation of a total score. Also, since the majority of women were included in one center, surgical ease was determined by a limited number of surgeons and should be interpreted as such.

## 5 CONCLUSION

Our study did not demonstrate non‐inferiority of UPA as a pretreatment compared with GnRHa. We had an underpowered study with a relatively small number of women. Confirmation of our findings is needed to make any final conclusions and based on our data we advise that larger studies, potentially of a superior study design, are carried out. Furthermore, fibroids in our study were large and these large fibroids in particular may benefit from volume reduction to facilitate a successful laparoscopic approach. From that perspective, volume reduction is important, since volume seems to be related to surgical ease and surgery time. Future studies should aim to confirm this.

## Supporting information


** **

** **

** **

** **

** **
