# Supplementary material for: Ulipristal acetate vs gonadotropin‐releasing hormone agonists prior to laparoscopic myomectomy (MYOMEX trial): Short‐term results of a double‐blind randomized controlled trial
Source: Acta Obstet Gynecol Scand. 2019 Sep 27;99(1):89–98. doi: 10.1111/aogs.13713 (PMC6973004; doi:10.1111/aogs.13713)
Supplement: Supplementary file 1 [file AOGS-99-89-s001.docx]

**Appendix S1– Outcome measures**

The primary outcome was intra-operative blood loss measured by using an uniform ‘intra-operative blood loss measurement’ protocol designed for this study. At the end of the surgery, all visible blood and irrigation fluid was removed from the abdominal cavity by applying extensive Trendelenburg and anti-Trendelenburg position until all blood and fluid was removed. The intra-operative blood loss was estimated measuring the quantity of blood in the suction bottle at the end of surgery minus the volume of irrigation fluid used during surgery. Time required for various steps was carefully measured during the procedure: time of surgery (i.e. start abdominal incision until suturing skin of abdomen), time of enucleation (i.e. start enucleation until complete resection of the fibroid) and time of suturing (i.e. time of suturing myometrium). Surgeons were asked to fill-out a questionnaire immediately after surgery on intra-operative outcomes and surgical ease. This questionnaire contained questions on specific details on removed fibroids (e.g. quantity, location within the uterus, weight) and on different technical aspect of the procedure (e.g. clamping of uterine artery or ovarian vessels, morcellation, opening of uterine cavity, conversion).
A surgical assessment tool to assess surgical ease was developed by two gynecologists (JH, WH) with extensive experience with laparoscopic myomectomy. A total of ten questions were developed with questions on specific aspects of the procedure: difficulty of entire procedure, satisfaction with entire procedure, difficulty of finding cleavage planes, difficulty of morcellation, consistency and grip of the fibroids removed, ease of stitching, grip of barbed sutures in the myometrium, bleeding tendency of the tissue and the anatomical result. For each answer, a 3- or 5-points scale was used depending on the type of question. All surgeons completed the assessment tool immediately after the procedure.
Transvaginal ultrasounds to assess uterine and fibroid volume were performed at baseline, after pre-treatment and six weeks post-operatively. Hemoglobin levels were assessed at baseline, after pre-treatment, within 48 hours after surgery and six weeks post-operatively.

*Patient questionnaires*Women received digital online secured validated questionnaires at baseline and after pre-treatment on menstrual blood loss (PBAC) ^1^, general health (Euro-QoL, Shaw and SF-36) ^2-4^, sexual functioning (FSFI and FSDS) ^5, 6^, menopausal symptoms ^7^ and quality of life at baseline (UFS-QOL) ^8^, after pre-treatment, six weeks and six months post-operatively.

**References**

1. Higham JM, O'Brien PM, Shaw RW. Assessment of menstrual blood loss using a pictorial chart. Br J Obstet Gynaecol. 1990;97:734-9.

2. EuroQol G. EuroQol--a new facility for the measurement of health-related quality of life. Health Policy. 1990;16:199-208.

3. Ware JE, Jr. SF-36 health survey update. Spine (Phila Pa 1976). 2000;25:3130-9.

4. Shaw RW, Brickley MR, Evans L, Edwards MJ. Perceptions of women on the impact of menorrhagia on their health using multi-attribute utility assessment. Br J Obstet Gynaecol. 1998;105:1155-9.

5. Derogatis LR, Rosen R, Leiblum S, Burnett A, Heiman J. The Female Sexual Distress Scale (FSDS): initial validation of a standardized scale for assessment of sexually related personal distress in women. J Sex Marital Ther. 2002;28:317-30.

6. Rosen R, Brown C, Heiman J, Leiblum S, Meston C, Shabsigh R, et al. The Female Sexual Function Index (FSFI): a multidimensional self-report instrument for the assessment of female sexual function. J Sex Marital Ther. 2000;26:191-208.

7. Oldenhave A, Jaszmann LJ, Everaerd WT, Haspels AA. Hysterectomized women with ovarian conservation report more severe climacteric complaints than do normal climacteric women of similar age. Am J Obstet Gynecol. 1993;168:765-71.

8. Spies JB, Coyne K, Guaou Guaou N, Boyle D, Skyrnarz-Murphy K, Gonzalves SM. The UFS-QOL, a new disease-specific symptom and health-related quality of life questionnaire for leiomyomata. Obstet Gynecol. 2002;99:290-300.
