# Supplementary material for: Ulipristal acetate vs gonadotropin‐releasing hormone agonists prior to laparoscopic myomectomy (MYOMEX trial): Short‐term results of a double‐blind randomized controlled trial
Source: Acta Obstet Gynecol Scand. 2019 Sep 27;99(1):89–98. doi: 10.1111/aogs.13713 (PMC6973004; doi:10.1111/aogs.13713)
Supplement: Supplementary file 2 [file AOGS-99-89-s002.docx]

**Appendix S2 - Post-operative complications and serious adverse events**

A total of six post-operative complications were reported, four in the ulipristal acetate group and two in the GnRHa group. One patient pre-treated with ulipristal acetate developed temporary fever of unknown cause and one patient developed an ileus. Both patients recovered quickly after adequate treatment. One patient in the ulipristal acetate was admitted to the hospital due to severe dyspnea, caused by a pneumothorax caused by endometriosis in the thoracic cavity (catamenial pneumothorax). This event was reported as a serious adverse event. One patient who received ulipristal acetate was admitted to the hospital due to severe vaginal bleeding three weeks after surgery which stopped spontaneously. This resulted in a hemoglobin level of 3.6 mmol/L for which she received three packed cells. This was also reported as a serious adverse event. In patients pre-treated with GnRHa, two complications occurred: one wound infection which improved spontaneously and one patient received a re-operation one day after surgery due to persistent abdominal pains and a drop in hemoglobin level. During second look laparoscopy, it became clear that there was no active bleeding in the abdomen and it was concluded that the drop in hemoglobin level possibly could be explained by dilution. The patient recovered without additional interventions.
