# Supplementary material for: Ulipristal acetate vs gonadotropin‐releasing hormone agonists prior to laparoscopic myomectomy (MYOMEX trial): Short‐term results of a double‐blind randomized controlled trial
Source: Acta Obstet Gynecol Scand. 2019 Sep 27;99(1):89–98. doi: 10.1111/aogs.13713 (PMC6973004; doi:10.1111/aogs.13713)
Supplement: Supplementary file 3 [file AOGS-99-89-s003.docx]

**Appendix S3 – Mediation analyses**

**Method**

A post-hoc analyses was performed to assess the role of the weight of fibroids removed as a possible mediating variable for the relation between pre-treatment to which patient was randomized and blood loss. The product of coefficients approach as implemented in M-plus version 7 was used to decompose the total effect of pre-treatment on blood loss in a direct and indirect effect.

**Result**

The geometric mean (GM) of intra-operative blood loss was found to be 2.41 (95% CI 1.18 to 1.83) times higher after pre-treatment with ulipristal acetate when compared to GnRHa. A post-hoc mediation analysis showed that this effect could be partly explained by an indirect effect of pre-treatment on blood loss via weight of fibroid (ratio of GM: 1.53, 95% CI 1.07 to 2.17; p = 0.018). The direct effect that together with this indirect effect makes up the total effect was not significant (ratio of GM: 1.58, 95% CI 0.92 to 2.69; p=0.093). Other possible intermediates such as vascularity were not recorded in all patients.
