# Supplementary material for: Ulipristal acetate vs gonadotropin‐releasing hormone agonists prior to laparoscopic myomectomy (MYOMEX trial): Short‐term results of a double‐blind randomized controlled trial
Source: Acta Obstet Gynecol Scand. 2019 Sep 27;99(1):89–98. doi: 10.1111/aogs.13713 (PMC6973004; doi:10.1111/aogs.13713)
Supplement: Supplementary file 4 [file AOGS-99-89-s004.docx]

**Table S1 – Inclusions per participating center**

| **Participating center** | **Ulipristal acetate** | **Leuprolide acetate** | **Total** |
| --- | --- | --- | --- |
| 1 | 19^#^ | 16^a^ | 35 |
| 2 | 3 | 3 ^a^ | 6 |
| 3 | 0 | 0 | 0 |
| 4 | 2 | 3 | 5 |
| 5 | 1 | 2 | 3 |
| 6 | 0 | 0 | 0 |
| 7 | 0 | 0 | 0 |
| 8 | 1^#^ | 1 | 2 |
| 9 | 4^#^ | 0 | 4 |

^a^One patient in this group did not undergo laparoscopic myomectomy (see Figure 1)

Sensitivity analyses for intra-operative blood loss (log-transformed) comparing center 1 to center 2-9:

Ulipristal acetate: 95%CI for difference -0.53 to 0.04, *P*=0.094

GnRHa: 95% CI for difference -0.49 to 0.36, *P*=0.752
