# Supplementary material for: Ulipristal acetate vs gonadotropin‐releasing hormone agonists prior to laparoscopic myomectomy (MYOMEX trial): Short‐term results of a double‐blind randomized controlled trial
Source: Acta Obstet Gynecol Scand. 2019 Sep 27;99(1):89–98. doi: 10.1111/aogs.13713 (PMC6973004; doi:10.1111/aogs.13713)
Supplement: Supplementary file 5 [file AOGS-99-89-s005.docx]

|  | **All fibroids (n=54)** | | | **Fibroids ≤ 8cm (n=29)** | | | **Fibroids ˃ 8cm (n=25)** | | |
| --- | --- | --- | --- | --- | --- | --- | --- | --- | --- |
|  | **UPA (n=29)** | **GnRHa (n=25)** | ***P* value** | **UPA (n=13)** | **GnRHa (n=16)** | ***P* value** | **UPA (n=16)** | **GnRHa (n=9)** | ***P* value** |
| **Pre-operative parameters** |  |  |  |  |  |  |  |  |  |
| Mean diameter of largest fibroid (cm; mean±SD)  *Baseline*  *3 months*  *Change from baseline to 3 months in % (median, IQR)* | 8.5 ± 1.9  8.4 ± 2.3  -3.6% (-15.5 to 10.4) | 7.4±1.6  5.8 ± 2.1  -14.6% (-40.7 to -5.6) | **0.035**  **0.003** | 6.7±0.9  6.6±1.5  -1.7% (-16.4 to 14.8) | 6.4±1.0  4.8±1.7  -12.4% (-43.3 to -6.9) | 0.423  **0.023** | 9.9±1.2  9.6±2.1  -6.0% (-15.5 to 7.3) | 9.1±0.7  6.9±1.8  -20.6 (-30.8 to 4.0) | 0.118  0.084 |
| Total fibroid volume planned for resection (cm^3^;median, IQR)  *Baseline*  *3 months*  *Change from baseline to 3 months in % (median, IQR)* | 316.3 (184.7 to 462.6)  319.4 (163.0 to 506.4)  -7.2% (-35.5 to 54.1) | 246.0 (130.2 to 344.3)  105.9 (54.4 to 195.9)  -38.4% (-71.5 to -19.3) | 0.133  **0.001** | 166.2 (108.9 to 233.2)  171.8 (97.5 to 320.0)  -5.1% (-31.4 to 72.2) | 153.9 (86.3 to 244.9)  57.4 (46.5 to 149.3)  -33.2% (-74.1 to 20.9) | 0.914  **0.005** | 450.7 (361.2 to 649.2)  473.1 (247.2 to 652.7)  -17.5% (-40.3 to 21.4) | 394 (328-490)  171.8 (98.5 to 413.3)  -49.0% (-66.0 to -15.1) | 0.357  0.065 |
| Uterine volume (cm^3^; median, IQR)  *Baseline*  *3 months*  *Change from baseline to 3 months in % (median, IQR)* | 530.5 (392.8 to 774.5)  598.2 (284.6 to 830.6)  -6.4% (-24.3 to 51) | 421.2 (327.5 to 819.8)  272.1 (180.4 to 508.8)  -26.1% (-63.4 to 4.2) | 0.510  **0.020** | 387.7 (162.0 to 619.7)  284.6 (238.0 to 664.5)  +3.8% (-21.2 to 50.9) | 381.4 (324.5 to 500.5)  226.9 (123.4 to 386.9)  -47.2% (-67.4 to -12.1) | 0.619  **0.006** | 681.5 (462.8 to 823.7)  667.7 (512.0 to 1175.8)  -6.4% (-34.3 to 68.3) | 514.6 (370.7 to 928.8)  480.0 (239.0 to 806.5)  +4.2 (-57.8 to 26.1) | 0.770  0.861 |
| I**ntra-operative and post-operative parameters** |  |  |  | **UPA (n=11)** | **GnRHa (n=14)** |  | **UPA (n=15)** | **GnRHa (n=9)** |  |
| Intra-operative blood loss (ml; median, IQR) | 525 (348-1025) | 280 (100-500) | **0.002**^a,b^ | 400 (300-1000) | 175 (50-313) | **0.005**^a^ | 610 (350-1300) | 500 (263-675) | 0.298^a^ |
| Total surgery time (minutes; median, IQR) | 188 (132-231) | 125 (100-175) | **0.023**^a,c^ | 177 (115-233) | 123 (98-171) | 0.190^a^ | 190 (140-230) | 147.0 (106-191) | 0.120^a^ |
| Suturing time (min; median, IQR) | 42 (29-51) | 25 (14-40) | **0.009** | 45 (33-70) | 15 (12-35) | 0.003 | 38 (27-46) | 37 (25-50) | 0.862 |
| Number of fibroids removed (n,%)   - 1 fibroid - ≥2 fibroids | 21 (81.0)  5 (19.0) | 19 (83.0)  4 (17.0) | 0.868 | 8 (72.7)  3 (27.3) | 10 (71.4)  4 (28.6) | 1.000 | 13 (86.7)  2 (13.3) | 9 (100.0)  0 (0.0) | 0.511 |
| Weight of fibroids removed (gram; median, IQR) | 349 (185-561) | 140 (61-272) | **0.001** | 183 (100-300) | 96 (53-153) | **0.049** | 495 (329-597) | 222 (74-367) | **0.005** |

**Table S2 – Subanalysis for fibroids smaller or equal to 8 cm and fibroids larger than 8 cm**

^a^Unpaired *t*-test performed on log transformed variable intra-operative blood loss and total surgery time
^b^After correction for confounder ‘mean diameter of largest fibroid’: *P*=0.011
^c^After correction for confounder ‘mean diameter of largest fibroid’: *P*=0.053
